# CoNiO_2_/Co_4_N Heterostructure Nanowires Assisted Polysulfide Reaction Kinetics for Improved Lithium–Sulfur Batteries

**DOI:** 10.1002/advs.202104375

**Published:** 2021-12-11

**Authors:** Jun Pu, Wenbin Gong, Zhaoxi Shen, Litong Wang, Yagang Yao, Guo Hong

**Affiliations:** ^1^ Institute of Applied Physics and Materials Engineering University of Macau Avenida da Universidade Taipa Macau SAR 999078 China; ^2^ School of Physics and Energy Xuzhou University of Technology Xuzhou 221018 China; ^3^ National Laboratory of Solid State Microstructures College of Engineering and Applied Sciences Jiangsu Key Laboratory of Artificial Functional Materials Collaborative Innovation Center of Advanced Microstructures Nanjing University Nanjing 210093 China; ^4^ Division of Nanomaterials and Jiangxi Key Lab of Carbonene Materials Suzhou Institute of Nano‐Tech and Nano‐Bionics Nanchang Chinese Academy of Sciences Nanchang 330200 China; ^5^ Department of Physics and Chemistry Faculty of Science and Technology University of Macau, Avenida da Universidade Taipa Macau SAR 999078 China

**Keywords:** CoNiO_2_/Co_4_N heterostructures, heterointerface, Li–S batteries, nanowires, reaction kinetics

## Abstract

The “shuttle effect” of soluble polysulfides and slow reaction kinetics hinder the practical application of Li–S batteries. Transition metal oxides are promising mediators to alleviate these problems, but the poor electrical conductivity limits their further development. Herein, the homogeneous CoNiO_2_/Co_4_N nanowires have been fabricated and employed as additive of graphene based sulfur cathode. Through optimizing the nitriding degree, the continuous heterostructure interface can be obtained, accompanied by effective adjustment of energy band structure. By combining the strong adsorptive and catalytic properties of CoNiO_2_ and electrical conductivity of Co_4_N, the in situ formed CoNiO_2_/Co_4_N heterostructure reveals a synergistic enhancement effect. Theoretical calculation and experimental design show that it can not only significantly inhibit “shuttle effect” through chemisorption and catalytic conversion of polysulfides, but also improve the transport rate of ions and electrons. Thus, the graphene composite sulfur cathode supported by these CoNiO_2_/Co_4_N nanowires exhibits improved sulfur species reaction kinetics. The corresponding cell provides a high rate capacity of 688 mAh g^−1^ at 4 C with an ultralow decaying rate of ≈0.07% per cycle over 600 cycles. The design of heterostructure nanowires and graphene composite structure provides an advanced strategy for the rapid capture–diffusion–conversion process of polysulfides.

## Graphical abstract

Uniform CoNiO_2_/Co_4_N heterostructure nanowires are prepared by a controlled hydrothermal and nitriding process. The in situ heterostructure with the continuous interface combine the strong polysulfide adsorption of CoNiO_2_ and the high electrical conductivity of Co_4_N. It not only effectively alleviates the “shuttle effect”, but also greatly improves the reaction dynamics of the battery.

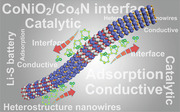

## 1 Introduction

Lithium–sulfur (Li–S) batteries have attracted much attention because of their much higher theoretical capacity (1672 mAh g^−1^) and energy density (2567 Wh kg^−1^) than those of conventional Li‐ion batteries, such as LiCoO_2_–graphite and LiFePO_4_–graphite.^[^ ^1^, ^2^ ^]^ However, the intermediates polysulfides (Li_2_S*_n_*, *n* = 4–8) can dissolve in the electrolyte and deposit on the counter electrode, resulting in “shuttle effect”. It will consume electroactive species, reducing Coulombic efficiency and capacity. Large amounts of polysulfides react with Li anode disproportionation, destroying the stability of the electrode interface, which greatly increases the threat of Li dendrites.^[^ ^3^, ^4^ ^]^ In addition, the poor electron conductivity of sulfur and slow reaction kinetics seriously hinder the rapid start‐up and response of Li–S batteries.^[^ ^5^ ^]^

In order to tackle the above‐mentioned issues, various efforts have been made in exploring new electrode structures and materials. Dispersing active sulfur into porous carbon matrix to form composite cathode is one of the main strategies.^[^ ^6^, ^7^, ^8^ ^]^ Among the carbon based materials, graphene has attracted extensive attention. Its excellent conductivity and large specific surface area are used as electronic network to reduce the sulfur interface resistance and physical barrier to inhibit the diffusion of polysulfides, respectively.^[^ ^8^ ^]^ Unfortunately, some inherent problems still exist in the graphene–sulfur (G–S) composite architectures. Nonpolar carbon surface has a poor affinity for polar hydrophilic polysulfides. Such weak capture ability results in limited cyclic stability and high sulfur loading.^[^ ^9^ ^]^ Functional groups (such as hydroxyl, carboxyl) modified graphene can enhance the adsorption of polysulfides. However, it will reduce the conductivity of the material and increase the complexity of preparation.^[^ ^10^, ^11^ ^]^ Moreover, the aggregation and stacking properties of graphene also need to be emphasized, which may cause unnecessary obstacles to ion diffusion.^[^ ^12^ ^]^

The combination of polar transition metal compounds (TMCs, such as oxides, nitrides) with graphene has proved to be an effective strategy to inhibit “shuttle effect” and enhance conversion of Li_2_S*_n_*.^[^ ^13^ ^]^ However, most TMCs have only one or two functions, which are relatively single and unable to integrate all the advantages. For instance, some metal oxides have strong polysulfide adsorption, while their poor electrical conductivity is not conducive to rapid charge transfer and will lead to severe electrode passivation.^[^ ^2^, ^13^, ^14^ ^]^ In contrast, metal nitrides with metal properties exhibit weak polysulfide affinity, resulting in a significant reduction in confinement effect and catalytic activity.^[^ ^15^, ^16^ ^]^ Recently, the design of TMC materials with different functions into a unified heterojunction structure has become a research focus to alleviate the above problems. For example, Luo et al. prepared heterophase V_2_O_3_–VN hollow structure.^[^ ^17^ ^]^ The multifunctional heterostructure was proved to be beneficial to chemical anchoring and catalytic conversion of polysulfides. Similarly, Xiao and co‐workers developed ZnS–SnS heterojunction with uniform cubic morphology to suppress the “shuttle effect”.^[^ ^18^ ^]^ Afterward, Wu et al. designed a twinborn holey Nb_4_N_5_–Nb_2_O_5_ heterostructure to combine the merits of electronic conducting (Nb_4_N_5_) with polysulfide adsorption (Nb_2_O_5_).^[^ ^19^ ^]^ Although these heterojunctions achieve good cathode performance through synergy effect, the large structure and size (usually above 100 nm in size) limit the overall energy density improvement.^[^ ^15^ ^]^ This is because these “inactive” additives do not provide additional reaction capacity, only surface layers play the role of adsorption and catalysis.^[^ ^13^ ^]^ Excessive usage can increase the overall weight of the battery. As a result, the design of heterojunction morphology is very important.

It can be seen that in addition to conductivity, adsorption and catalysis, another feature of an ideal G–S cathode additive is the minimization of the amount of material used. Ultrafine nanoparticles or ultrathin nanosheets are powerful structural designs, but neither of them can well alleviate the issue of graphene agglomeration. Previous studies have shown that the introduction of 1D structure can effectively alleviate the agglomeration of graphene layers.^[^ ^20^, ^21^ ^]^ More importantly, the high aspect ratio shape has a lower penetration threshold and is easy to form a continuous conductive network.^[^ ^22^, ^23^ ^]^ Therefore, it will be a promising challenge to construct heterojunctions with different characteristics for Li_2_S*_n_* on TMC nanowires as graphene based sulfur cathode medium.

**Figure 1:**
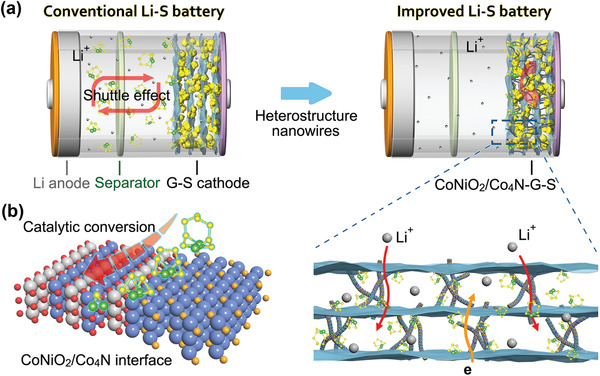
a) A comparison of the conventional Li–S battery with “shuttle effect” and the CoNiO_2_/Co_4_N based improved cell. b) Schematic illustration of interface mechanism of heterostructure and polysulfides.

In this study, a graphene composite matrix supported by CoNiO_2_/Co_4_N heterostructure nanowires was prepared from the point of view of physical properties and structure. The in situ formed heterostructure greatly enhanced the polysulfide reaction kinetics and reduced the “shuttle effect” of Li–S batteries (**Figure** **1**a). We have studied the interface and synergistic effect of the CoNiO_2_/Co_4_N heterostructure theoretically and experimentally. On one hand, it combined the strong polysulfide anchoring ability of CoNiO_2_ and the excellent electrical conductivity of Co_4_N. On the other hand, as the active site, the heterogeneous interface had stronger ability of ion diffusion and polysulfide transformation (Figure 1b). As a result, the unique CoNiO_2_/Co_4_N–G–S cathode delivered high initial discharge capacity of 1198 mAh g^−1^ at 0.2 C and 688 mAh g^−1^ at 4 C, and favorable cycling stability over 600 cycles with an ultraslow capacity decay of ≈0.07% per cycle.

## 2 Results and Discussion

**Figure 2:**
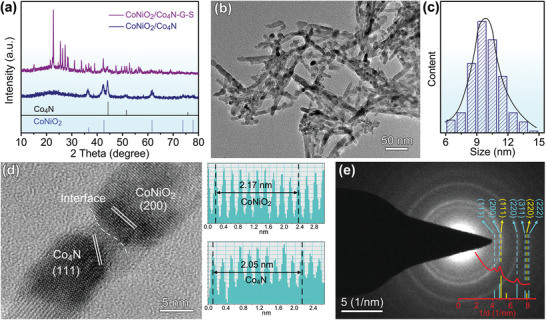
Phase characterization of CoNiO_2_/Co_4_N heterostructure nanowires. a) XRD patterns. b,c) TEM image and diameter distribution. d) HRTEM result and corresponding lattice spacing. e) SAED pattern and rotational integral.

The phase and composition information of as‐prepared samples was first characterized by X‐ray diffraction (XRD). As shown in Figure S1a in the Supporting Information, the pattern of precursor was well matched with standard diffraction peaks of NiCo_2_O_4_ (JCPDS No. 20‐0781), without any contamination observed. After nitridation, a mixed phase appeared (**Figure** **2**a), which meant the precursor had undergone a significant phase transition. The diffraction peaks at 36.8°, 42.8°, and 61.7° could be readily assigned to CoNiO_2_ phase (JCPDS No. 10‐0188), and the diffraction peaks at 44.1° and 51.7° were indexed to cubic Co_4_N structure (JCPDS No. 15‐0806).^[^ ^24^, ^25^ ^]^ This binary mixing state was further investigated by X‐ray photoelectron spectra (XPS). It was not difficult to see from Figure S2a in the Supporting Information that the Co 2p_3/2_ orbit could be separated into three peaks, indicating Co existed in more than one chemical state.^[^ ^26^ ^]^ The peaks at 779.3 and 782.4 eV were well attributed to the Co—Co and Co—N bonds, respectively.^[^ ^24^, ^26^, ^27^ ^]^ The existence of Co—Co metal bond was due to the similar atomic structure of Co_4_N and metallic Co (0), and the N element was located in the middle of the unit cell.^[^ ^27^, ^28^ ^]^ The peak located at 780.8 eV was assigned to the Co—O bond, which was derived from CoNiO_2_.^[^ ^29^ ^]^ In the N 1s XPS spectra (Figure S2b, Supporting Information), the peak at 398.2 eV verified the existence of Co—N.^[^ ^30^, ^31^ ^]^ The energy‐dispersive X‐ray spectroscope (EDS) result (Figure S3, Supporting Information) also demonstrated the incorporation of N. The oxidation states of Ni 2p_3/2_ orbit in Figure S2c in the Supporting Information could be deconvolution derived from divalent and trivalent Ni ions.^[^ ^32^ ^]^ These results indicated the formation of CoNiO_2_/Co_4_N heterostructure, which combined the characteristics of CoNiO_2_ and Co_4_N concurrently.^[^ ^31^ ^]^ The XRD of the CoNiO_2_/Co_4_N–G–S composite (Figure 2a) confirmed the presence of layered graphene (Figure S1b, Supporting Information) and orthorhombic sulfur (Figure S1c, Supporting Information), which could be used as polysulfide host and active materials, respectively. From thermogravimetric (TG) analysis in Figure S4 in the Supporting Information, it could be seen that the sulfur content in CoNiO_2_/Co_4_N–G–S, CoNiO_2_–G–S, Co_4_N–G–S, and G–S were 66.7%, 66.5%, 66.9%, and 66.6%, respectively. More importantly, the characteristic peaks of CoNiO_2_/Co_4_N had not changed significantly, indicating that this preparation method would maintain the stability of heterostructure.

The transmission electron microscopy (TEM) and scanning electron microscopy (SEM) images observed in Figure 2b and Figure S5 in the Supporting Information show an overall morphology of the CoNiO_2_/Co_4_N sample. It reveals that the heterojunction material presented a uniform nanowire‐like structure with an average diameter of 8–12 nm (Figure 2c). These nanowires were overlapped with each other and would form a mixed 3D conductive framework with layered graphene (Figure S6, Supporting Information). When graphene was combined with CoNiO_2_/Co_4_N, CoNiO_2_ and Co_4_N samples, their Barrett–Emmett–Teller (BET) surface areas were up to 621.3, 665.6, 620.2 m^2^ g^−1^, respectively (Figure S7a–c, Supporting Information). Similar specific surface area ensured uniform loading of sulfur and comparability of corresponding Li–S batteries. Meanwhile, the partially graphitized nature of as‐obtained graphene based composite was confirmed by Raman technology in Figure S7d–f in the Supporting Information. Two prominent peaks at about 1352 and 1587 cm^−1^ corresponded to the characteristic D and G bands of carbon structure, respectively. Figure 2d depicts a high‐resolution TEM (HRTEM) image with clear lattice fringes for further revealing as‐prepared CoNiO_2_/Co_4_N heterostructure. The lattice spacing of 0.217 and 0.205 nm corresponded to the (200) plane of NiO and (111) plane of CoNiO_2_, respectively. A higher magnification and clearer HRTEM image is shown in Figure S8 in the Supporting Information. The distinct heterointerface could be easily observed. The resulting interface would be used not only as an active site for the adsorption and conversion of soluble polysulfides, but also as a channel for rapid Li‐ion diffusion, thus greatly improving the electrochemical performance of Li–S batteries.^[^ ^5^, ^32^ ^]^ The rotational integral from selected area electron diffraction (SAED) pattern (Figure 2e) were consistent with XRD result, which further indicated the formation of CoNiO_2_/Co_4_N. XPS characterization revealed that the ratio of CoNiO_2_ to Co_4_N in the heterojunction was ≈1.8:1.

**Figure 3:**
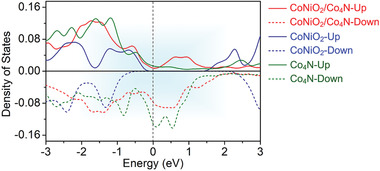
DOS of optimized geometries of CoNiO_2_/Co_4_N, CoNiO_2_, and Co_4_N.

The electronic conductivities of CoNiO_2,_ Co_4_N, and CoNiO_2_/Co_4_N were calculated via partial density of states (DOS) plots. As shown in **Figure** **3**, pristine CoNiO_2_ was a typical semiconductor, while Co_4_N had metallic characteristics. It was noteworthy that after engineering the in situ heterojunction, not only the band structure of Co_4_N remained intact, but also the band structure of the heterostructure crossed the Fermi level, indicating the excellent conductive properties of CoNiO_2_/Co_4_N. Such conductivity would also facilitate the rapid transfer of electrons and polysulfide conversion.^[^ ^33^ ^]^

**Figure 4:**
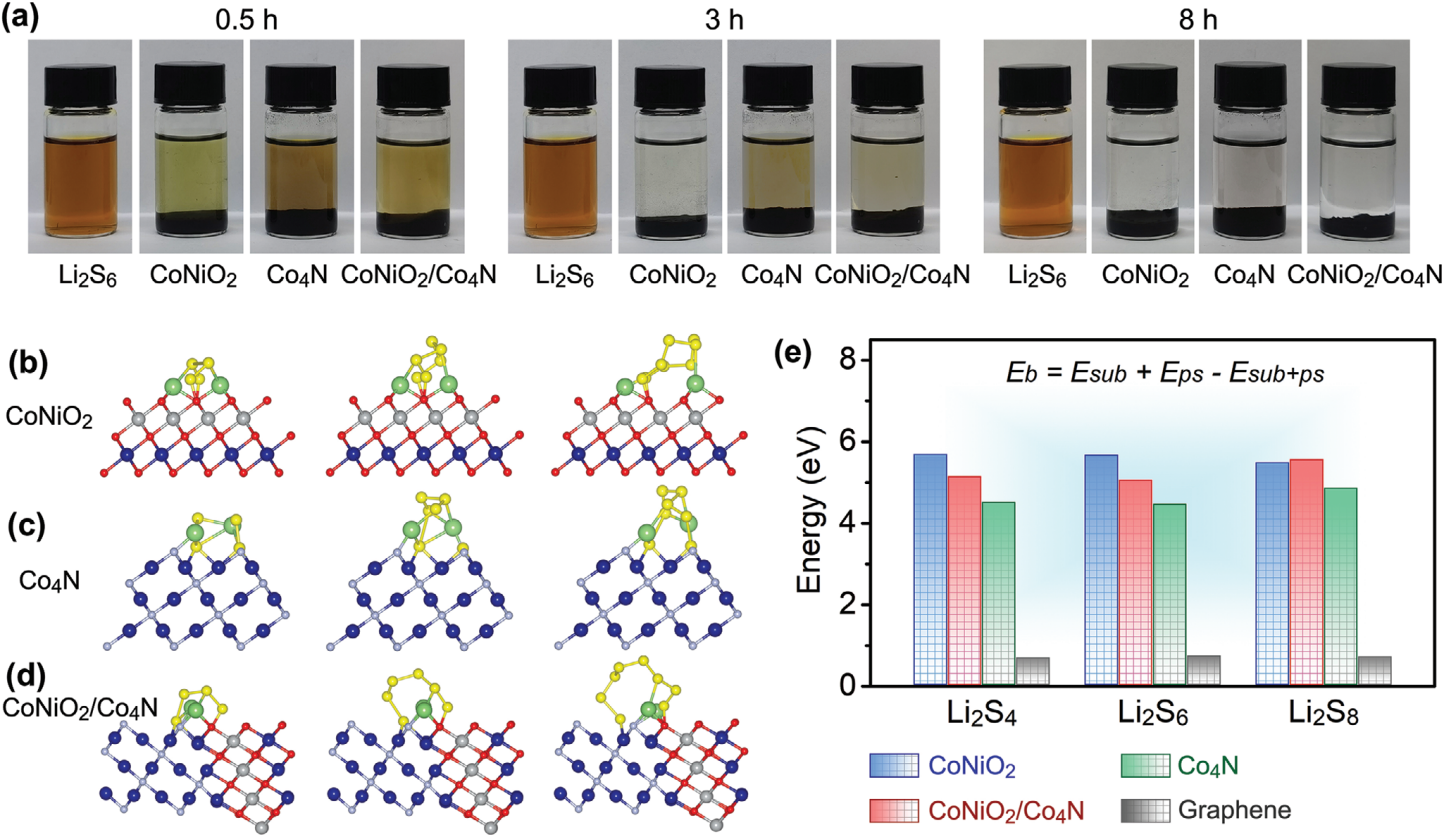
a) Visual Li_2_S_6_ adsorption test over time. Optimized geometries of Li_2_S_6_ on different substrates: b) CoNiO_2_ (200) surface, c) Co_4_N (111) surface, and d) corresponding CoNiO_2_/Co_4_N heterojunction interface. e) Contrast diagram of calculated adsorption energies.

Subsequently, a series of visual adsorption experiments were designed to investigate the anchoring behavior of as‐prepared samples to soluble polysulfides. Equal amounts of CoNiO_2_, Co_4_N, CoNiO_2_/Co_4_N were added to the brown‐yellow solution of Li_2_S_6_, respectively, and their changes with time were observed (**Figure** **4**). All the samples could decolorize Li_2_S_6_ solution in varying degrees within 0.5 h, indicating that they all had certain adsorption characteristics for polysulfides. After 3 h, the color of Li_2_S_6_ solution with adding pure CoNiO_2_ and heterostructure had changed significantly, especially the former, which was almost completely clarified. However, the Co_4_N sample did not reach the transparent effect until 8 h later, indicating that the anchoring ability of Li_2_S_6_ is in the order of CoNiO_2_, CoNiO_2_/Co_4_N, and Co_4_N, from strong to weak. This difference could also be confirmed by density functional theory (DFT) (Figure 4b–e). As can be seen from Figure 4e, the adsorption energies of Li_2_S_6_ on CoNiO_2_, Co_4_N and CoNiO_2_/Co_4_N interface were calculated to be 5.69, 4.48, and 4.48 eV respectively, which was basically consistent with the results of the above visual test. Moreover, Li_2_S_4_ showed similar results. Interestingly, for Li_2_S_8_, the heterojunction revealed a higher binding energy than monomeric CoNiO_2_ or Co_4_N, implying tighter anchoring and rapid adsorption. All these adsorption values were much higher than those on the surface of pure graphene. This might be because the interaction between the carbon and polysulfides was only weak van der Waals forces (Figure S9, Supporting Information).^[^ ^34^, ^35^ ^]^ In contrast, the adsorption of polysulfides on Ni—Co substrates resulted from chemical bonding (Figure 4b–d). It should be noted that these bonds did not significantly change the structure of polysulfide molecules, which meant that the irreversible decomposition of Li_2_S*_n_* can be avoided.^[^ ^35^ ^]^

The XPS results in Figure S2 in the Supporting Information further analyzed the interaction between the CoNiO_2_/Co_4_N and Li_2_S_6_. After interacting with Li_2_S_6_, an additional peak appeared at 780.2 eV of the Co 2p_3/2_ spectrum, indicating the formation of Co—S bond.^[^ ^36^, ^37^ ^]^ The shift of the N 1s orbital to higher binding energy (blue‐shift) indicated the transfer of electrons from the Co—N to the terminal sulfur.^[^ ^38^, ^39^ ^]^ The Ni 2p_3/2_ peak shifted slightly toward the lower binding energy (red‐shift), which was similar to previous literatures, meaning that a small amount of electrons transferred from the polysulfide to the Ni atom.^[^ ^40^, ^41^, ^42^ ^]^ Such tight affinity of CoNiO_2_/Co_4_N heterostructure to polysulfides would not only effectively improve the anchoring ability, but also promote charge transfer and facilitate the rapid conversion of sulfur species.

**Figure 5:**
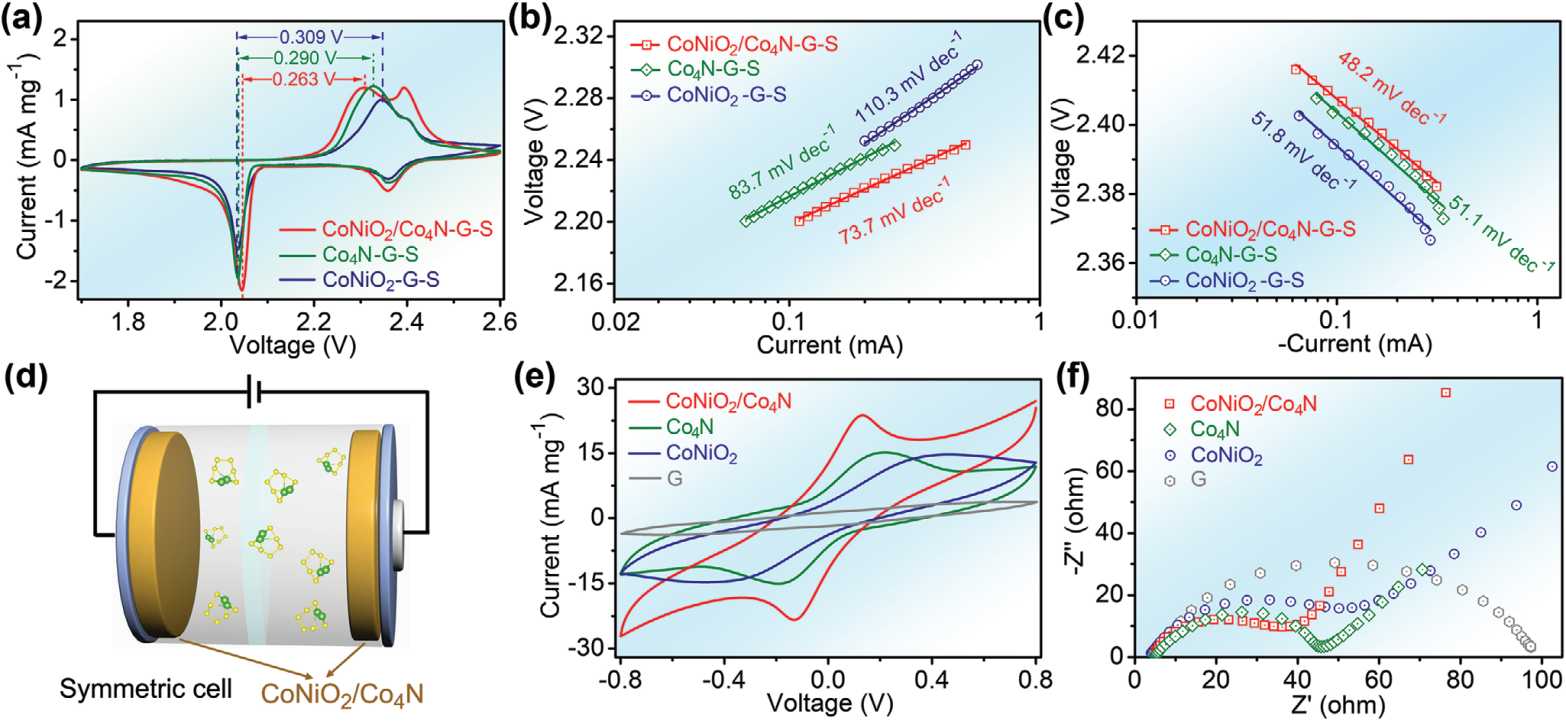
Verification of catalytic properties of CoNiO_2_/Co_4_N heterostructure. a) CV curves of the CoNiO_2_/Co_4_N–G–S, CoNiO_2_–G–S, and Co_4_N–G–S cathodes in a potential window of 1.7–2.6 V at 0.1 mV s^−1^. Corresponding Tafel plots and fitted slope values for b) the oxidation peak and c) the reduction peak at ≈2.4 V from three CV curves. c) Local magnification of charge–discharge curves of different cathodes. d) Schematic diagram of symmetrical cell. e) CV contrast profiles of Li_2_S_6_ symmetric cells. f) Nyquist plots of different symmetric cells.

In order to explore the effect of CoNiO_2_/Co_4_N heterostructure on polysulfide reaction kinetics, the fully assembled Li–S batteries were first tested by cyclic voltammogram (CV). In general, the strong anodic peak at about 2.3–2.4 V is related to the conversion of short‐chain Li_2_S_2_/Li_2_S to long‐chain polysulfides and elemental sulfur.^[^ ^23^, ^43^ ^]^ Two cathodic peaks (≈2.35 and ≈2.05 V) are attributed to the reduction of sulfur to long‐chain polysulfides and subsequent formation of Li_2_S_2_/Li_2_S.^[^ ^3^, ^37^ ^]^ As shown in **Figure** **5**a, all CV curves showed typical redox peaks for Li–S batteries and no additional peaks appeared, indicating that none of the three samples obstructed normal polysulfide reactions. The CoNiO_2_/Co_4_N exhibited the lowest anode potential and the highest cathode potential, and the voltage polarization was as low as 0.263 V. Even the CoNiO_2_–G–S electrode with highest polarization showed less polarization than pure graphene without any additives (Figure S10, Supporting Information). Considering the conductivity of CoNiO_2_ and graphene, the polarization difference fully indicated that CoNiO_2_ had a good catalytic function of polysulfides.^[^ ^44^ ^]^ Meanwhile, the corresponding Tafel plots and slopes of all peaks were also calculated to quantify the catalytic activities (Figure 5b,c and Figure S11, Supporting Information). Evidently, the CoNiO_2_/Co_4_N–G–S electrode exhibited the lowest slopes at the oxidation of Li_2_S and the reduction of sulfur to long‐chain sulfur species, implying the greatest improvement in the kinetics.^[^ ^45^, ^46^ ^]^ For the cathodic peak at ≈2.0 V, the slope of CoNiO_2_/Co_4_N–G–S was not much different from that of the lowest Co_4_N–G–S electrode, indicating that the charge in the heterostructure could also be rapidly transformed for this reaction.

The conversion kinetics of as‐prepared samples was more obvious in symmetrical cell based on Li_2_S_6_ catholyte (Figure 5d).^[^ ^4^ ^]^ From the CV curves in Figure 5e, except for graphene, the other electrodes showed two distinct redox peaks and high response current, indicating easy conversion between Li_2_S*_n_* and Li_2_S_2_/Li_2_S.^[^ ^47^ ^]^ The redox peak of CoNiO_2_/Co_4_N sample provided the highest intensity and smallest polarization, which was in good agreement with the electrochemical impedance spectroscopy (EIS) results (Figure 5f) for symmetric cells. The charge transfer resistance of symmetrical cell increased in the order of CoNiO_2_/Co_4_N<Co_4_N<CoNiO_2_<graphene, suggesting that the introduction of heterostructure resulted in the obvious improvement in polysulfide conversion kinetics.

**Figure 6:**
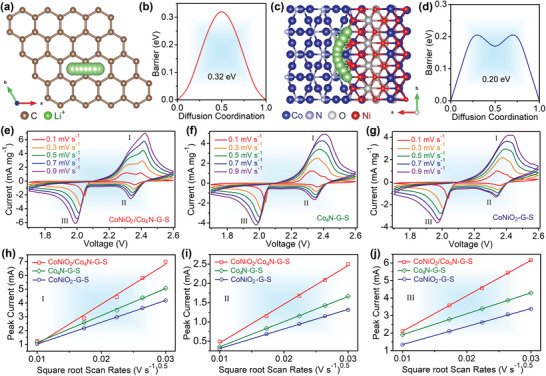
The illustration of Li ion migration pathways and corresponding barriers profles: a,b) graphene and c,d) CoNiO_2_/Co_4_N heterostructure. e–g) CV of as‐obtained three electrodes at various scan rates. h–j) The linear fits of the peak currents from CV curves.

In addition to adsorption and conductivity, Li‐ion diffusion is another important factor affecting the dynamics of Li–S system.^[^ ^48^ ^]^ To better understand the S_8_/S_8_^2−^ redox properties of CoNiO_2_/Co_4_N nanowires, DFT calculated the migration of Li‐ion at the heterostructure interface and graphene surface (**Figure** **6**a–d). The Li‐ion diffusion barrier of graphene exhibited higher barrier with peak value of 0.32 eV, while the peak value of CoNiO_2_/Co_4_N was as low as 0.321 eV. The low diffusion energy meant that the ions migrate effectively on the heterojunction substrate, which ensured the strong interfacial ion transfer kinetics.^[^ ^49^ ^]^ Moreover, CV analysis of CoNiO_2_/Co_4_N–G–S, Co_4_N–G–S and CoNiO_2_–G–S electrodes in the range of 0.1–0.9 mV s^−1^ were carried out in Figure 6e–g. It could be seen that all the reaction peak currents were linear with the square root of the scanning rate. According to Randles–Sevcik equation (see Supporting Information for details), the diffusivity of Li‐ion can be simply judged by the slope of the line.^[^ ^45^, ^50^, ^51^ ^]^ As shown in Figure 6h–j, all slopes of CoNiO_2_/Co_4_N displayed the highest values based on linear fitting, indicating a faster ion diffusion capability. Overall, combined with the analysis of CV and symmetric cells, it was not difficult to find that CoNiO_2_/Co_4_N heterostructure did have the most suitable catalytic activity, which would help to avoid the formation of “dead sulfur”.^[^ ^18^ ^]^

**Figure 7:**
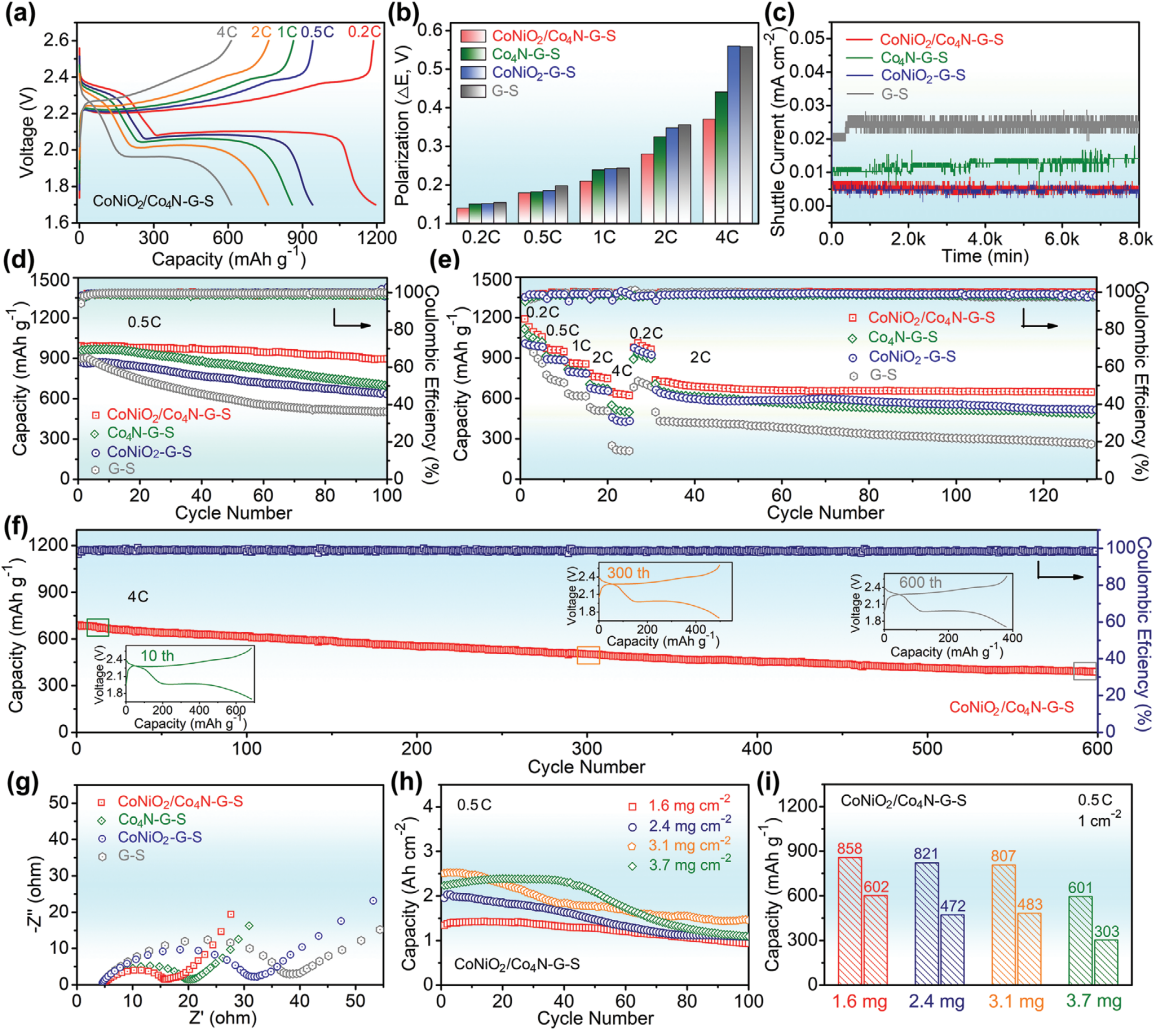
Electrochemical performance of CoNiO_2_/Co_4_N–G–S and other control cathodes. a) Charge–discharge profiles of CoNiO_2_/Co_4_N–G–S at various current densities. b) Comparing the potential polarizations of different electrodes. c) Shuttle current test. d) Cyclic properties of different cathodes at 0.5 C. e) The rate conversion and the subsequent constant current cycle. f) Cyclic stability of the CoNiO_2_/Co_4_N–G–S electrode at high rate. g) EIS curves at 20th cycle. (h,i) Cyclic performance of CoNiO_2_/Co_4_N–G–S with different sulfur loading at 0.5 C.

The above theoretical calculations and experimental designs have preliminarily evaluated the advantages of CoNiO_2_/Co_4_N heterostructure. In order to verify the performance improvement of the actual Li–S battery, a series of electrochemical tests were carried out. **Figure** **7**a shows the voltage‐capacity diagrams at various C‐rates from 0.2 to 4 C. All profiles showed two distinct discharge platforms and a wide charging platform, which were consistent with CV curves. It indicated that the CoNiO_2_/Co_4_N–G–S electrode could complete the reversible conversion of polysulfides well even at the high rate of 4 C.^[^ ^52^ ^]^ At 0.2, 0.5, 1, 2, and 4 C, capacities of 1198, 941, 860, 765, and 618 mAh g^−1^ were achieved, respectively. In this voltage range, CoNiO_2_/Co_4_N itself only exhibited a capacity of ≈13 mAh g^−1^ (Figure S12, Supporting Information), much lower than that of CoNiO_2_/Co_4_N–G–S, indicating the efficient utilization of sulfur in the composite electrode. Furthermore, with the increase of rate, the polarization (Δ*E*) value and the increase range of the heterostructure based cell were the lowest among all the comparison samples, indicating that CoNiO_2_/Co_4_N provided the most significant improvement in cell dynamics.

To further verify the inhibition effect of the prepared samples on the “shuttle effect”, shuttle current tests were performed. The typical Li–S battery was discharged at 0.5 C to 2.38 V, and then adjusted to constant potential mode. The value of stabilized current can be called shuttle current.^[^ ^3^, ^53^ ^]^ As shown in Figure 7c, the G–S electrode unsurprisingly showed the maximum shuttle current. On the contrary, the values of CoNiO_2_/Co_4_N–G–S, and CoNiO_2_–G–S were the lowest, and the value of Co_4_N–G–S was in the middle, indicating the strong polysulfide adsorption of the former two. This was basically consistent with the above theoretical calculation and visual experiment.

Figure 7d compares the cycling performance of different cathodes (1.0 mg cm^−2^) at 0.5 C. The CoNiO_2_/Co_4_N–G–S electrode provided a better cycling stability, maintaining 896 mAh g^−1^ at 100th cycle with a high Coulombic efficiency of 99.2%. The retention rate of CoNiO_2_/Co_4_N–G–S as high as 90.2% was much higher than 72.8% of Co_4_N–G–S, 76.5% of CoNiO_2_–G–S, and 56.4% of G–S. Surprisingly, CoNiO_2_ had a high adsorption for polysulfides, but showed unsatisfactory cycle stability, which might be related to its poor electrical conductivity.^[^ ^32^, ^54^ ^]^ The rate performances of all the samples were evaluated by current density conversion and cycling (Figure 7e). With the increase of the cycling rate, the decrease of CoNiO_2_/Co_4_N–G–S capacity was obviously smaller than that of the other three electrodes. After switching back to 0.2 C, its capacity could be recovered to 997 mAh g^−1^, indicating the superior reversibility and rapid charge–discharge of the CoNiO_2_/Co_4_N–G–S cathode. In particular, during the following 100 cycles of 2 C, the heterostructure sample showed a capacity decrease of 12.8%, which was the lowest among all samples. In contrast, the CoNiO_2_–G–S, Co_4_N–G–S, and G–S cells lost almost 23.9%, 31.4%, and 46.7%, respectively. Figure 7f shows a higher rate (4 C) and longer cycle test of CoNiO_2_/Co_4_N–G–S. Since there was no previous cycle, its initial capacity was up to 688 mAh g^−1^. The Coulombic efficiency rapidly increased from 95.7% in the first cycle to 99.6% in the 15 cycles. The reversible capacity retained a high value of 389 mAh g^−1^ at 600th with a stabilized Coulombic efficiency of nearly 99%, which corresponds to a low decaying rate of ≈0.07% per cycle. Even after 1100 cycles, it still retained considerable capacity, with a corresponding decline rate of only 0.056% per cycle (Figure S13, Supporting Information). The charge–discharge curve of CoNiO_2_/Co_4_N–G–S remained stable without any obvious change in polarization, suggesting a homogeneous deposition of insoluble Li_2_S_2_/Li_2_S products without over thick accumulation of insulating sulfides.^[^ ^55^ ^]^ Such high rate and low capacity attenuation were superior to most of the previous and recent literatures (Figure S14, Supporting Information).^[^ ^56^ ^−^ ^64^ ^]^

The EIS spectra of all electrodes after cyclic testing are displayed in Figure 7g. Due to excellent electrical conductivity, CoNiO_2_/Co_4_N–G–S and Co_4_N–G–S showed approximately small charge transfer resistance (*R*_ct_). The slightly larger *R*_ct_ of CoNiO_2_–G–S further proofed its catalytic ability of polysulfide conversion. In comparison with G–S, all the other electrodes showed much smaller *R*_ct_ at high‐frequency region. Moreover, the heterostructure demonstrated a steeper straight line at low‐frequency, suggesting faster ion migration that was basically agree with the foregoing analysis.^[^ ^65^ ^]^ This boosted reaction kinetics not only benefited from the modification of the conductivity of the material, but also from the catalytic effect on polysulfides in the liquid phase.

The construction of high loading electrode is another challenge facing the practical application of high energy density Li batteries.^[^ ^34^ ^]^ Figure 7h shows the cyclic performance of CoNiO_2_/Co_4_N–G–S cathode with different sulfur loading at 0.5 C. Areal capacities of 1.37, 1.97, 2.50, and 2.20 mAh cm^−2^ were obtained for the cathodes with 1.6, 2.4, 3.1, and 3.7 mg cm^−2^ loadings, respectively, and the corresponding capacity was 858, 821, 807, and 601 mAh g^−1^ (Figure 7i). Due to the acceleration of polysulfide redox kinetics, all high loading cells showed small voltage polarization (Figure S15, Supporting Information). After 100 cycles, the impressive capacity of 602, 472, 483, and 303 mAh g^−1^ were still maintained.

**Figure 8:**
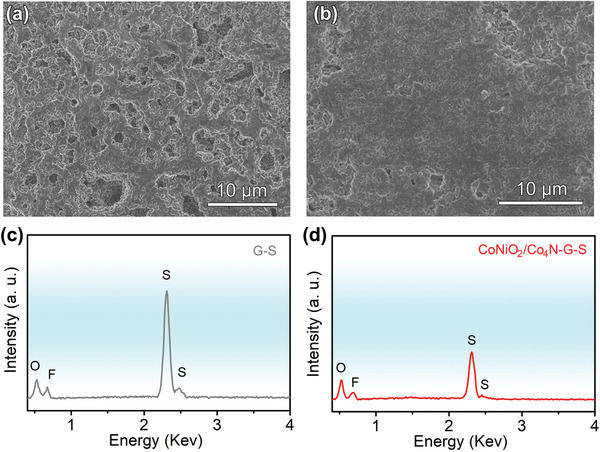
SEM of cycled Li–S anodes: a) G–S and b) CoNiO_2_/Co_4_N–G–S. Corresponding EDS results of c) G–S and d) CoNiO_2_/Co_4_N–G–S.

Post cycling analysis could further prove that heterojunction alleviated the “shuttle effect”. **Figure** **8**. shows the morphology and elemental analysis of the Li anode after cycling. From Figure. 8a,b, it was not difficult to see that the surface of the Li anode in G–S cell presented a porous structure and was covered with Li dendrites and “dead Li”, which is similar to previous literatures.^[^ ^38^, ^66^ ^]^ The rough surface might result from the disproportionation reaction caused by the diffusion of a large number of soluble polysulfide compounds toward the anode. The deposited sulfur species modulating the growth morphology of metallic Li.^[^ ^38^ ^]^ In contrast, for CoNiO_2_/Co_4_N–G–S cell, the Li anode surface was bright without obvious structural damage. A small amount of polysulfide was involved in strengthening the construction of solid–electrolyte interphase film, which made the metal interface more stable and smooth during cycling.^[^ ^67^ ^]^ The corresponding EDS spectra (Figure. 8c,d) show the same results. The content of sulfur on the Li surface of CoNiO_2_/Co_4_N–G–S battery was much less than that of G–S battery, which meant that the CoNiO_2_/Co_4_N could effectively inhibit the diffusion of polysulfides and slow down the “shuttle effect”. In addition, the XRD analysis (Figure S16, Supporting Information) after cycling showed that the CoNiO_2_/Co_4_N heterojunction still maintained its original phase, indicating that its overall structure did not change significantly under Li–S electrochemical reactions. This excellent stability would greatly promote the long‐term operation of Li–S batteries. Such good electrochemical properties and post cycling analysis further corroborated the effectiveness of CoNiO_2_/Co_4_N heterostructure nanowires design.

## 3 Conclusions

In summary, to alleviate the polysulfide incompatibility and kinetic delay of graphene host of Li–S batteries, CoNiO_2_/Co_4_N heterostructure nanowires medium was successfully prepared. In this design, the nanowire structure would effectively avoid graphene stacking and agglomeration. The band structure of CoNiO_2_ was accurately tuned by introducing Co_4_N, which effectively improved the electrical conductivity. Benefiting from the interface effect, as‐obtained heterostructure also retained the higher polysulfide adsorption and electrocatalytic activity of CoNiO_2_, which greatly reduced the “shuttle effect”. Meanwhile, CoNiO_2_/Co_4_N–G–S cathode showed a stronger Li‐ion diffusion behavior. Therefore, the overall performance of cell was enhanced due to the alleviated polysulfide diffusion and improved electrochemistry reaction kinetics. As a result, the CoNiO_2_/Co_4_N based Li–S cell achieved high reversibility capacity of 1198 mAh g^−1^ at 0.2 C, high rate performance (688 mAh g^−1^ at 4 C), and excellent cyclic stability of 600 cycles with low capacity attenuation of ≈0.07% each cycle on average. The coupling mechanism of graphene supported by heterostructure nanowires will provide new insights and possibilities for the practical application of Li–S batteries in the future.

## 4 Experimental Section

### Preparation of CoNiO_2_/Co_4_N Heterogeneous Nanowires

The NiCo_2_O_4_ nanowire precursor was synthesized by simple hydrothermal‐calcination method: 3 mmol Ni(CH_3_COO)_2_·4H_2_O and 6 mmol Co(CH_3_COO)_2_·4H_2_O were added with 1.1 mmol cetyltrimethylammonium bromide (CTAB) and 18 mmol urea into 20 mL of deionized water with stirring to a homogeneous solution. The solution was transferred into 50 mL Teflon‐lined autoclave and maintained at 110 °C for 6 h. After cooling to room temperature, the precipitate was centrifuged and washed. The dried product was annealed at 350 °C for 3 h to obtain the NiCo_2_O_4_ nanowires. Subsequently, as‐prepared oxide precursor was nitrided at 350 °C for 1 h in a mixed atmosphere composed of NH_3_ (150 sccm) and Ar (100 sccm) to form CoNiO_2_/Co_4_N heterogeneous nanowires.

### Preparation of CoNiO_2_/Co_4_N–G–S Composite Cathode

Graphene was prepared by classic Hummers method and reduction at high temperature (1000 °C). After that, the graphene and CoNiO_2_/Co_4_N were mixed and dispersed in deionized water at a weight ratio of 2:1. After ultrasonic and stirring, the mixture was freeze‐dried and mechanically ground. The process of loading sulfur was similar to the CS_2_ dissolution method in the literature.^[^ ^6^ ^]^ As‐obtained CoNiO_2_/Co_4_N–G composite was wetted with sulfur solution (CS_2_/*N*‐methy‐pyrrolidinone (NMP), 1:1) and dried at 50 °C for 12 h. Note that the content of sulfur in this compound was controlled at 67%. For comparison experiments, other composite cathodes (such as CoNiO_2_–G–S, Co_4_N–G–S) were prepared by the same process.

### Materials Characterization

The morphology and size of the as‐prepared samples were conducted by SEM (Hitachi S‐8100) and TEM (Tecnai G^2^ F30). The SAED was performed with TEM system at high acceleration voltage of 200 kV. The elemental analyses were collected by XPS (Thermo Scientific K‐Alpha) and EDS (Thermo Fischer, F200x). XRD patterns were characterized for the crystal structure and crystallinity using a Shimadzu X‐ray‐6000 diffractometer with Cu K_*α*_ radiation. The Raman spectra were characterized by Labram HR‐800) with optical micrographs (Nikon A1). The BET surface area was obtained by N_2_ adsorption measurement on a Kubo X1000 equipment at 77 K. TG analysis was performed using NETZSCH ASAP2020 thermal analyzer.

### Visual Polysulfde Adsorption Test

Sulfur and Li_2_S (5:1 in molar ratio) were mixed and stirred in 1,2‐dimethoxyethane (DME) solvent to obtain a brown‐yellow Li_2_S_6_ solution (8.0 mmol L^−1^). Subsequently, the equivalent amount of CoNiO_2_/Co_4_N heterostructure, CoNiO_2_, and Co_4_N were added into the above Li_2_S_6_ solution (5.0 mL), respectively.

### Electrochemical Measurements

Typically, the CoNiO_2_/Co_4_N sample, Super P, and polyvinylidene fluoride (PVDF) binder were mixed in NMP solvent with a mass ratio of 8:1:1 to obtain cathode slurry. Then, this slurry was coated onto Al foil current collector using a film applicator. The loading of active sulfur was controlled to 1.0−3.7 mg cm^−2^ by adjusting the thickness of the coating. After vacuum drying, as‐prepared cathode, Celgard 2400 separator, and Li anode were assembled in coin cell and tested at room temperature. The electrolyte was LiTFSI (1 mol L^−1^) in 1,3‐dioxolane (DOL) and DME (1:1, volume ratio) with 2 wt% LiNO_3_. The ratios of electrolyte versus sulfur (E/S) were 15 µL mg^−1^ (for typical sulfur loading of 1.0 mg cm^−2^) and 10 µL mg^−1^ (for high sulfur loadings of 1.6–3.7 mg cm^−2^), respectively. CV and EIS were measured VSP potentiostat (Bio‐Logic Corp.) and CHI 760D electrochemical analyzer (ChenHua Corp.). The galvanostatic charge–discharge cycling was performed using a Land Battery Tester. The same assembly process was used for the comparison samples.

For symmetrical cells, two identical electrodes were fabricated by a method similar to previous literatures.^[^ ^4^, ^5^ ^]^ The weight ratio of each additive material (CoNiO_2_/Co_4_N, CoNiO_2_, Co_4_N, and graphene), conductive agent and PVDF was 7:2:1. Using the above conventional LiTFSI based electrolyte as the solvent, a 0.5 mol L^−1^ Li_2_S_6_ solution was prepared as the new electrolyte. The CV scan rate was 50 mV s^−1^.

### Theoretical Calculations

The optimize geometries and electronic properties of all the investigated structures in this study were calculated at the DFT. The heterojunction was constructed by combining the CoNiO_2_ (111) surface with Co_4_N (200) surface based on the experimental observations. Vienna ab initio simulation package (VASP) were employed in the simulations using the projector augmented wave (PAW) potentials with a plane wave cutoff of 500 eV. The Perdew Burke Ernzerhof (PBE) form of the exchange correlations functional was employed in the simulation. Spin‐polarized calculations were performed for all the structures except for the graphene. The *k*‐point meshes in the simulations were generated using the VASPKIT tool with the grid separation of 0.4 Å^−1^ for the geometry optimizations and 0.1 Å^−1^ for the DOS calculations. All the structures were optimized by using the conjugate gradient method, in which the convergence for total energy and interaction force was set to be 10^−4^ eV and 10^−3^ eV Å^−1^, respectively.

The binding strength *E*_b_ of Li_2_S_4_, Li_2_S_6_, and Li_2_S_8_ on the five investigated substrates were calculated as follows: *E*_b_ = (*E*_sub_ + *E*_ps_) − *E*_sub+ps_, where *E*_sub+ps_, *E*_ps_, and *E*_sub_ denote the calculated energies of the total adsorption system, adsorbates, and substrates, respectively. For evaluating the diffusion barrier of Li on the surfaces of graphene and heterojunction, the transitional state was located using the Nudged Elastic Band method.

## Conflict of Interest

The authors declare no conflict of interest.

## Supporting information

Supporting Information

## Data Availability

Research data are not shared.
