# Supplementary material for: CoNiO2/Co4N Heterostructure Nanowires Assisted Polysulfide Reaction Kinetics for Improved Lithium–Sulfur Batteries
Source: Adv Sci (Weinh). 2021 Dec 11;9(4):2104375. doi: 10.1002/advs.202104375 (PMC8811817; doi:10.1002/advs.202104375)
Supplement: Supplementary file 1 — Supporting Information [file ADVS-9-2104375-s001.pdf]

WILEY-VCH

ADVANCED SCIENCE
Open Access

Supporting Information

for *Adv. Sci.*, DOI: 10.1002/advs.202104375

$CoNiO_2$/Co_4N Heterostructure Nanowires Assisted Polysulfide Reaction Kinetics for Improved Lithium–Sulfur Batteries

Jun Pu, Wenbin Gong, Zhaoxi Shen, Litong Wang, Yagang Yao, and Guo Hong**

Supporting Information

$CoNiO_2$/Co_4N Heterostructure Nanowires Assisted Polysulfide Reaction Kinetics for Improved Lithium–Sulfur Batteries

Jun Pu, Wenbin Gong, Zhaoxi Shen, Litong Wang, Yagang Yao, and Guo Hong**

Dr. J. Pu, Z. Shen, L. Wang, Prof. G. Hong
Institute of Applied Physics and Materials Engineering, University of Macau Avenida da Universidade Taipa, Macau SAR 999078, China
E-mail: ghong@um.edu.mo

Prof. W. Gong
School of Physics and Energy, Xuzhou University of Technology, Xuzhou 221018, China

Prof. Y. Yao
National Laboratory of Solid State Microstructures, College of Engineering and Applied Sciences, Jiangsu Key Laboratory of Artificial Functional Materials, Collaborative Innovation Center of Advanced Microstructures, Nanjing University, Nanjing 210093, China
E-mail: ygyao2018@nju.edu.cn

Prof. Y. Yao
Division of Nanomaterials and Jiangxi Key Lab of Carbonene Materials, Suzhou Institute of Nano-Tech and Nano-Bionics, Nanchang, Chinese Academy of Sciences, Nanchang 330200, China

Prof. G. Hong
Department of Physics and Chemistry, Faculty of Science and Technology, University of Macau. Avenida da Universidade, Taipa, Macau SAR 999078, China

Experimental Section

Synthesis of $CoNiO_2$ and Co_4N:

For $CoNiO_2$: 4.5 mmol $Ni(CH_3COO)_2{\cdot}4H_2O$, 4.5 mmol $Co(CH_3COO)_2{\cdot}4H_2O$, 1.1 mmol cetyltrimethylammonium bromide (CTAB), and 18 mmol urea were added into 20 mL of deionized water. This homogeneous solution was transferred into 50 mL Teflon-lined autoclave and maintained at 110 °C for 6 h. After cooling to room temperature, the precipitate was centrifuged and washed. The dried product was annealed at 350 °C for 3 h to obtain the $CoNiO_2$.

For Co_4N: 9 mmol $Co(CH_3COO)_2{\cdot}4H_2O$, 1.1 mmol CTAB, and 18 mmol urea were added into 20 mL of deionized water with stirring. Above solution was transferred into 50 mL Teflon-lined autoclave and maintained at 110 °C for 6 h. After cooling to room temperature, the precipitate was centrifuged and washed. The dried product was annealed at 350 °C for 3 h. Subsequently, as-prepared oxide precursor was nitriding at 350 °C for 2 h in a mixed atmosphere composed of NH_3 (150 sccm) and Ar (100 sccm).

Randles-Sevcik Equation:

$$I_p = (2.69 \times 10^5) n^{1.5} S D^{0.5} C v^{0.5}$$

where I_p is the peak current, n is the number of electrons transferred, S is the active electrode area, C is the Li ion concentration and v is the scan rate], the Li ion diffusion coefficient (D) is positively correlated with the slope of the curve ($I_p/v^{0.5}$).[1,2] The higher the slope, the stronger the ion diffusion ability.[3,4].

Figures:

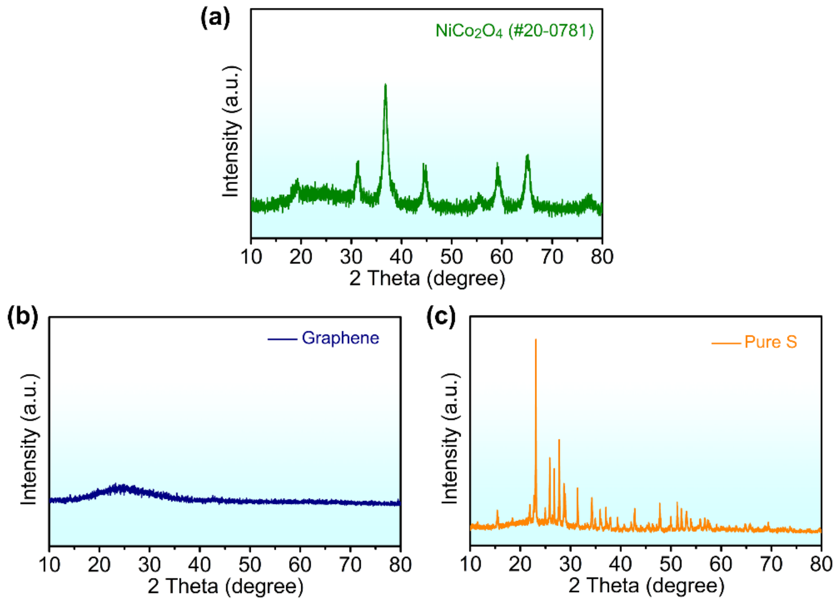

Figure S1. XRD patterns of (a) $NiCo_2O_4$ precursor, (b) graphene, and (c) pure sulfur.

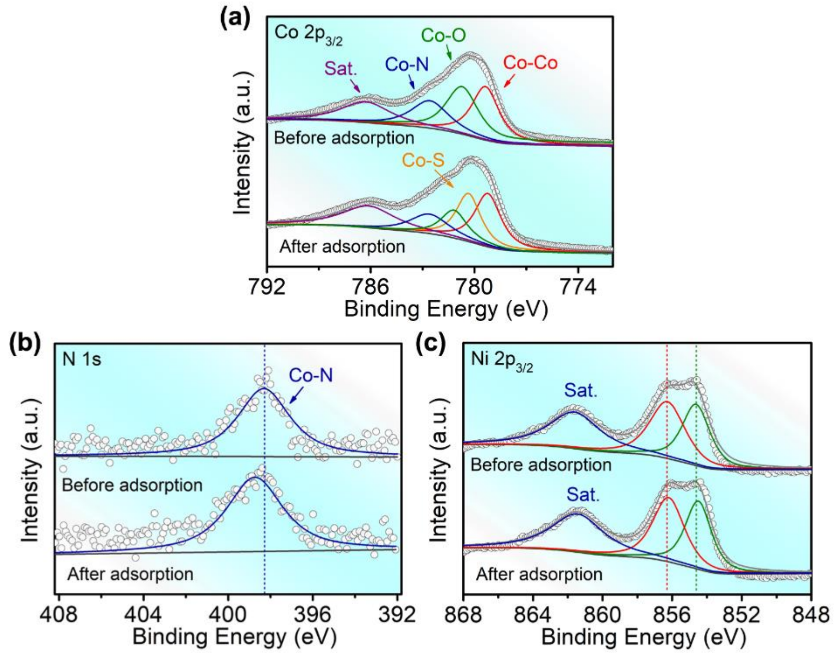

Figure S2. XPS of $CoNiO_2/Co_4N$ heterostructure before and after adsorption Li_2S_6: (a) Co $2p_{3/2}$, (b) N 1s, and (c) Ni $2p_{3/2}$.

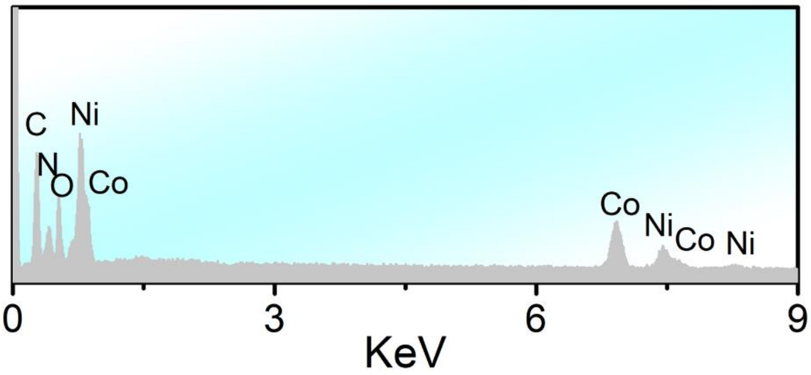

Figure S3. EDS of $CoNiO_2/Co_4N$ sample.

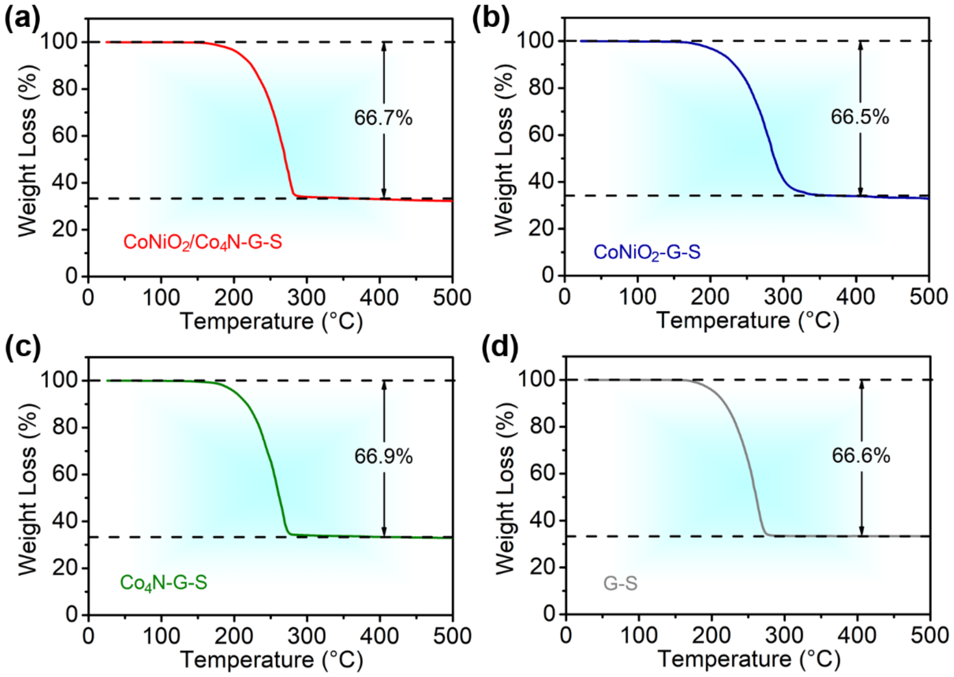

Figure S4. TGA curves of (a) $CoNiO_2/Co_4N$-G-S, (b) $CoNiO_2$-G-S, (c) Co_4N-G-S, and (d) G-S sapmles.

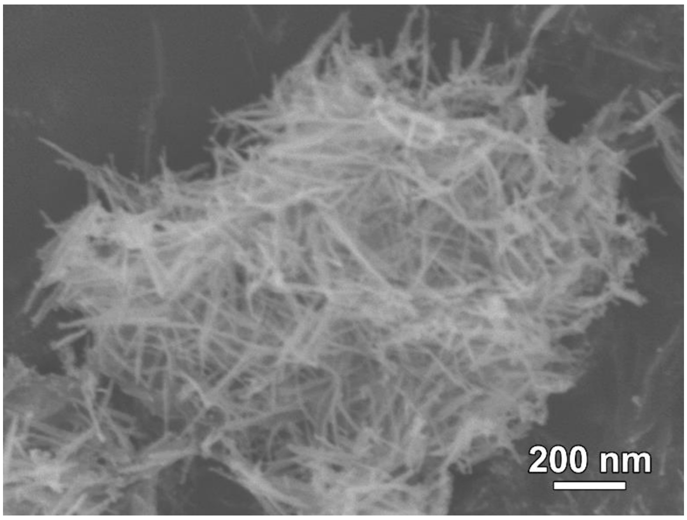

Figure S5. SEM image of $CoNiO_2/Co_4N$ heterostructure nanowires.

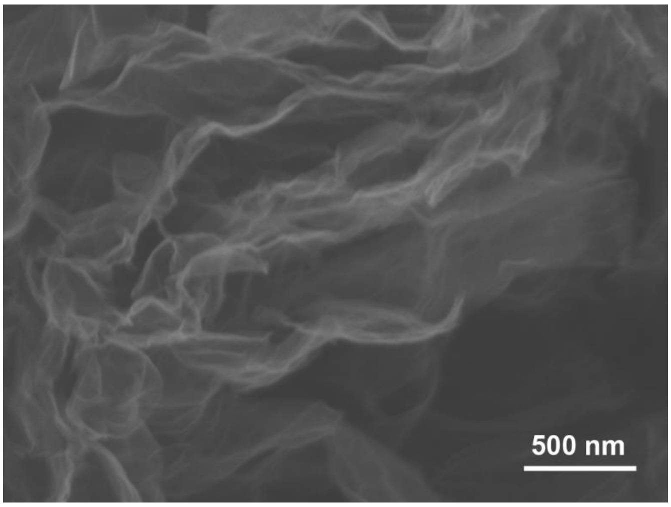

Figure S6. SEM image of graphene.

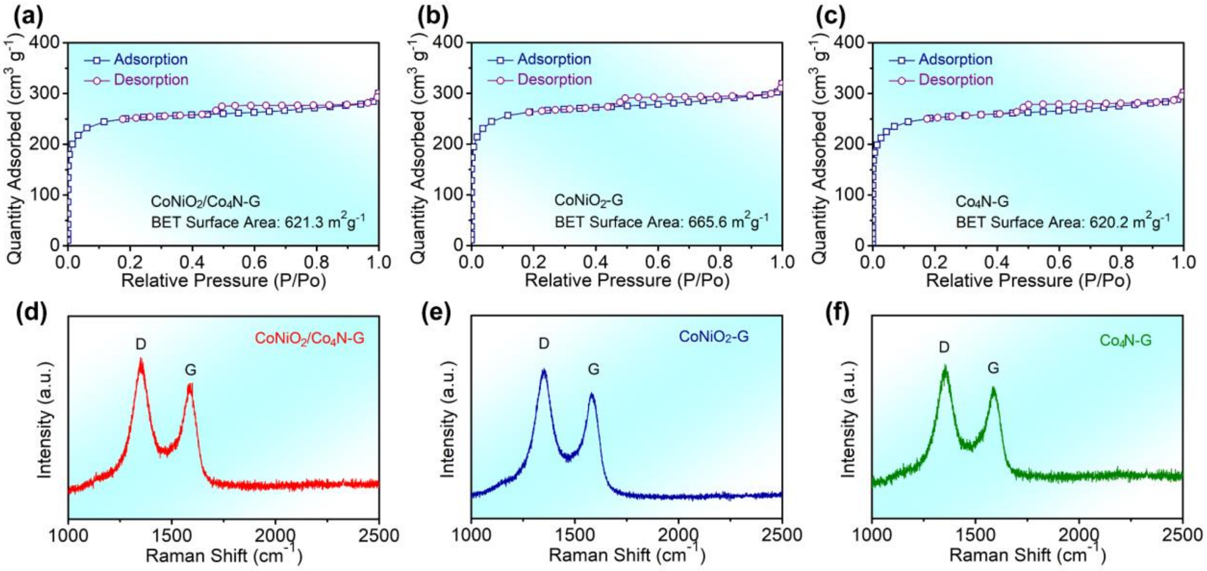

Figure S7. (a–c) N_2 sorption isotherms and (d–f) Raman spectra of the $CoNiO_2/Co_4N$-G, $CoNiO_2$-G, and Co_4N-G composites.

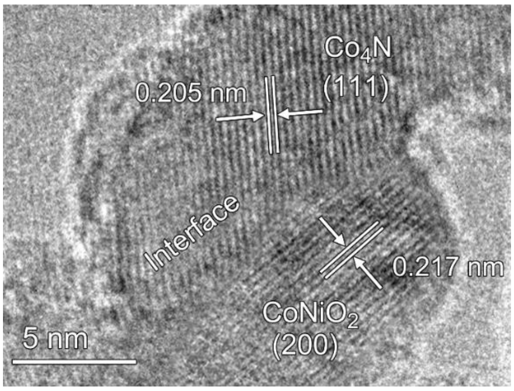

Figure S8. A locally amplified HRTEM image of $CoNiO_2/Co_4N$ heterostructure.

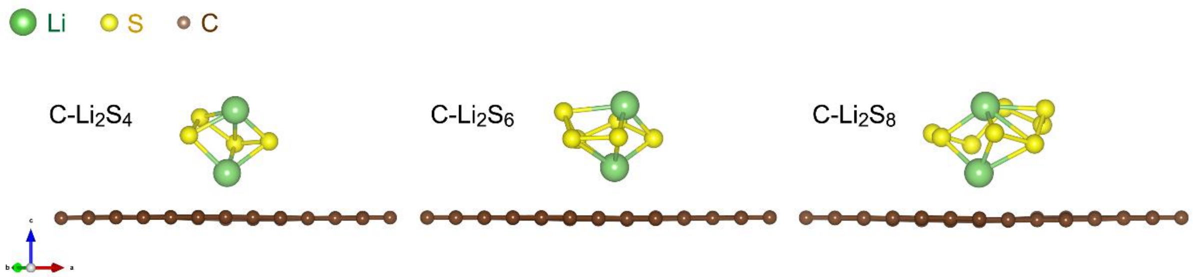

Figure S9. Polysulfides (Li_2S_4, Li_2S_6, Li_2S_8) adsorbed on the surface of graphene.

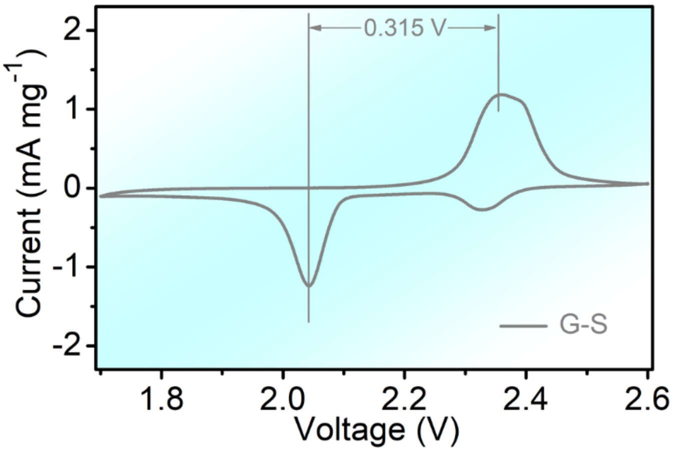

Figure S10. CV of G-S cathode at 0.1 mV s^{-1}.

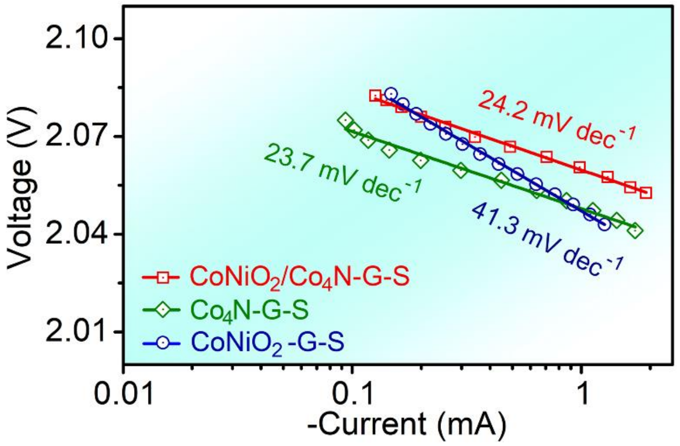

Figure S11. Tafel plots of the reduction peaks at about 2.05 V.

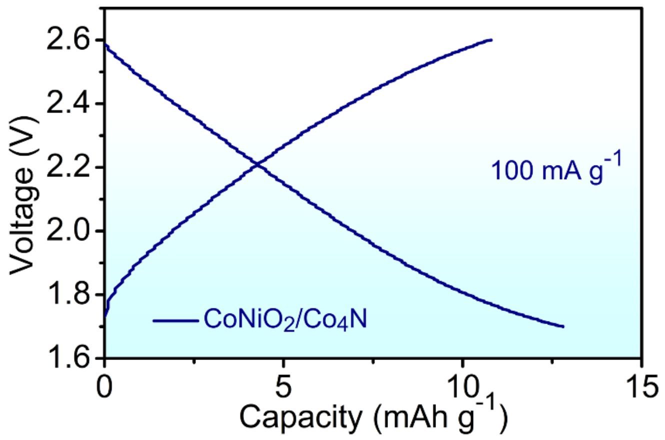

Figure S12. Galvanostatic discharge-charge curve of pure $CoNiO_2/Co_4N$ heterostructure nanowires.

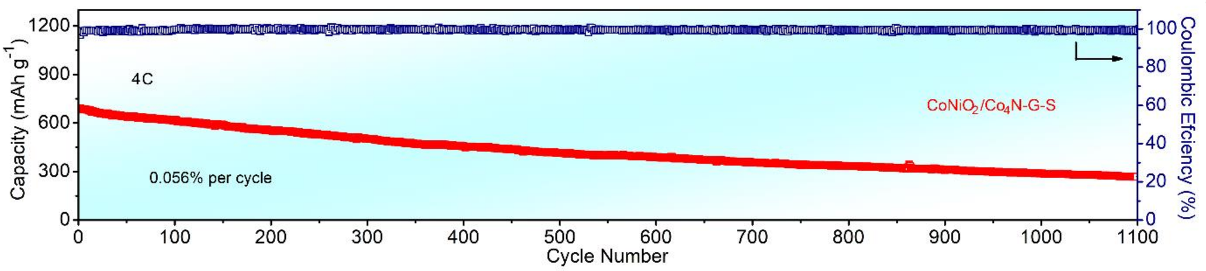

Figure S13. Long-term cycling life of $CoNiO_2/Co_4N$-G-S cathode at 4 C for 1100 cycles.

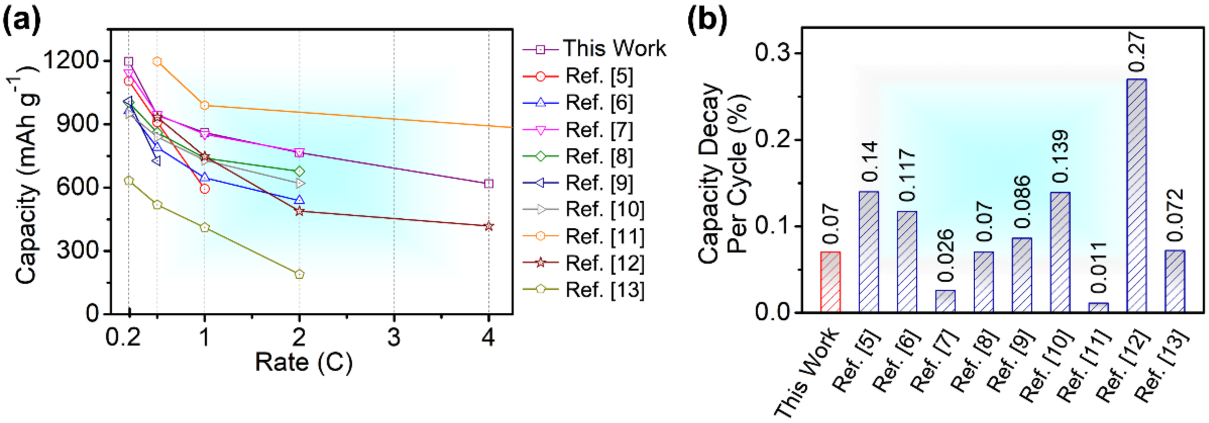

Figure S14. (a) Rate performance and (b) capacity decay per cycle of the $CoNiO_2/Co_4N$ heterostructure cathode compared to other reported Li–S batteries.[5–13]

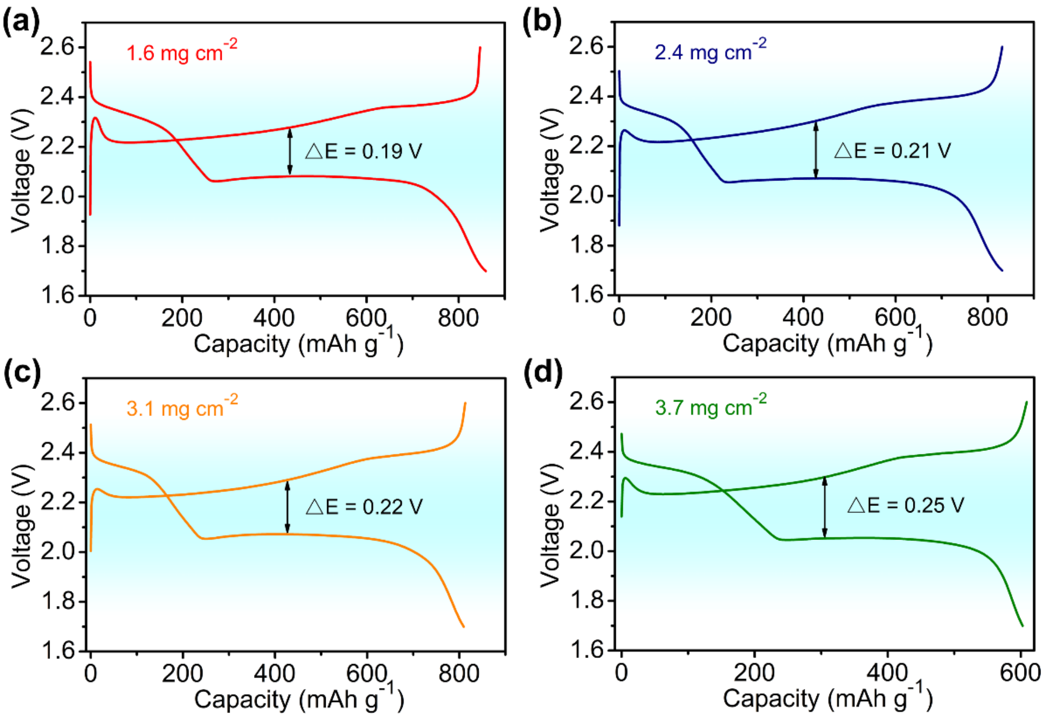

Figure S15. Galvanostatic discharge-charge curves of $CoNiO_2/Co_4N$-G-S with different sulfur high loading at 0.5 C.

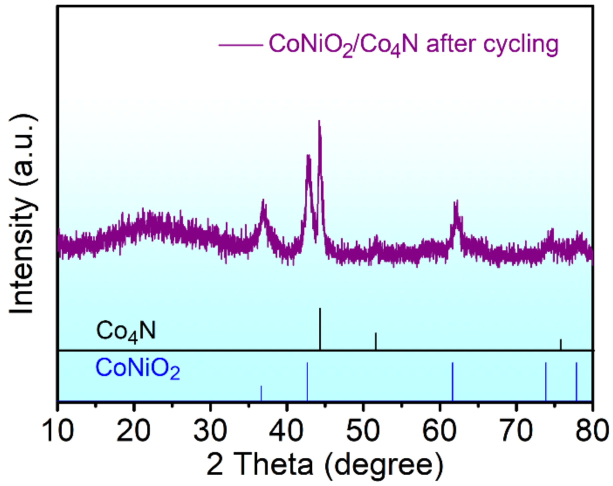

Figure S16. XRD of $CoNiO_2/Co_4N$ sample after cycling.

References

[1] H. Yamin, A. Gorenshtein, J. Penciner, Y. Sternberg, E. Peled, *J. Electrochem. Soc.*, **1988**, *135*, 1045.

[2] H. Yamin, J. Penciner, A. Gorenshtain, M. Elam, E. Peled, *J. Power Sources*, **1985**, *14*, 129.

[3] Z. Q. Ye, Y. Jiang, L. Li, F. Wu, R. J. Chen, *Adv. Mater.*, **2020**, *32*, 2002168.

[4] W. L. Li, J. Qian, T. Zhao, Y. S. Ye, Y. Xing, Y. X. Huang, L. Wei, N. X. Zhang, N. Chen, L. Li, F. Wu, R. J. Chen. *Adv. Sci.*, **2019**, *6*, 1802362.

[5] J. D. Shen, X. J. Xu, J. Liu, Z. B. Liu, F. K. Li, R. Z. Hu, J. W. Liu, X. H. Hou, Y. Z. Feng, Y. Yu, M. Zhu, *ACS Nano*, **2019**, *13*, 8986.

[6] M. Ma, L. Y. Cao, K. Yao, J. Y. Li, K. J. Kajiyoshi, J. F. Huang, *ACS Sustainable Chem. Eng.*, **2021**, *9*, 5315.

[7] W. Z. Bao, L. Liu, C. Y. Wang, S. Choi, D. Wang, G. X. Wang, *Adv. Energy Mater.*, **2018**, *9*, 1702485.

[8] D. B. Xiong, S. Z. Huang, D. L. Fang, D. Yan, G. J. Li, Y. P. Yan, S. Chen, Y. L. Liu, X. L. Li, Y. V. Lim, Y. Wang, B. B. Tian, Y. M. Shi, H. Y. Yang, *Small*, **2021**, *17*, 2007442.

[9] J. Y. Liu, M. F. Zhu, Z. H. Shen, T. L. Han, T. Si, C. Q. Hu, H. G. Zhang, *Small*, **2021**, *17*, 2103051.

[10] S. H. Shen, L. Huang, X. L. Tong, R. F. Zhou, Y. Zhong, Q. Q. Xiong, L. J. Zhang, X. L. Wang, X. H. Xia, J. P. Tu, *Adv. Mater.*, **2021**, *33*, 2102796.

[11] S. Q. Chen, X. D. Huang, H. Liu, B. Sun, W. K. Yeoh, K. F. Li, J. Q. Zhang, G. X. Wang, *Adv. Energy Mater.*, **2014**, *5*, 1301761.

[12] M. D. Zhang, C. Yu, C. T. Zhao, X. D. Song, X. T. Han, S. H. Liu, C. Hao, J. S. Qiu, *Energy Storage Mater.*, **2016**, *5*, 223.

[13] C. Y. Yan, W. Q. Li, X. J. Liu, M. Chen, X. Liu, X. M. Li, J. T. Zai, X. F. Qian, *ACS Appl. Mater. Interfaces*, **2021**, *13*, 48872.
